# Moderate Differences in Feeding Diets Largely Affect Motivation and Spatial Cognition in Adult and Aged but Less in Young Male Rats

**DOI:** 10.3389/fnagi.2018.00249

**Published:** 2018-08-15

**Authors:** Jovana Maliković, Daniel D. Feyissa, Ahmed M. Hussein, Harald Höger, Gert Lubec, Volker Korz

**Affiliations:** ^1^Department of Pharmaceutical Chemistry, University of Vienna, Vienna, Austria; ^2^Department of Zoology, Faculty of Science, Al-Azhar University, Assiut, Egypt; ^3^Core Unit of Biomedical Research, Division of Laboratory Animal Science and Genetics, Medical University of Vienna, Vienna, Austria; ^4^Department of Neuroproteomics, Paracelsus Medical University, Salzburg, Austria; ^5^Center for Brain Research, Medical University of Vienna, Vienna, Austria

**Keywords:** nutritional status, aging neuroscience, statistics, individuality, food reward responsivity

## Abstract

Nutrition can have significant effects on behavior and cognitive processes. Most of the studies related to this use extremely modified diets, such as high fat contents or the exclusion of distinct components needed for normal development and bodily homeostasis. Here we report significant effects of diets with moderate differences in compositions on food rewarded spatial learning in young (3–4 months), adult (6–7 months), and aged (17–18 months) rats. Young rats fed with a lower energy diet showed better performance only during aquisition of the spatial task when compared to rats fed with a standard diet. Adult rats (6–7 months) fed with a standard diet performed less well in the spatial learning task, than rats fed with lower energy diet. Aged rats fed with a lower energy diet (from 13 to 18 months of age) performed better during all training phases, as in a previous test when they were adult and fed with a standard diet. This difference could only be partly explained by lower motivation to search for food in the first test. Correspondingly, the variability of individual performance was significantly higher and increased over trials in adult rats fed with the standard diet as compared to adult rats fed with lower energy diet. Thus, moderate changes in feeding diets have large effects on motivation and cognition in elderly and less in young rats in a food rewarded spatial learning task. Therefore, nutrition effects upon food rewarded spatial learning and memory should be considered especially in aging studies.

## Introduction

Effects of caloric restriction upon cognition in aged rats have been reported previously (Adams et al., 2008; Rich et al., 2010; Kaptan et al., 2015). However, the results are controversial with beneficial (Gyger et al., 1992; Rich et al., 2010; Kaptan et al., 2015), no effects (Beatty et al., 1987; Bond et al., 1989; Hansalik et al., 2006) or impairments (Yanai et al., 2004) of spatial learning and memory. This may be related to different constraints.

First, most of the studies work with long-lasting food restriction, thus involuntary reduction of food consumption, high fat diet or by the exclusion of nutrients required for normal development and bodily homeostasis (Berrocal-Zaragoza et al., 2014; Boitard et al., 2014; Lépinay et al., 2015; De Luca et al., 2016; He et al., 2017). High fat diet and the unavailability of essential nutrients, however, are of clinical relevance and does not necessarily play a role in everyday life. Involuntary restriction causes chronic stress indicated by chronically elevated levels of corticosterone (Han et al., 2001; Pesic et al., 2010; MacDonald et al., 2014) which affects behavior (Duclos et al., 2009) and neuronal and synaptic plasticity (Pavlides et al., 1993) and may counteract possible beneficial effects of food restriction (Qiu et al., 2012). Mild chronic stress especially impairs spatial cognition in food rewarded tasks like the holeboard (Conrad, 2010).

Moreover, long-lasting food restriction enhances general activity in aged rats (Todorovic et al., 2018) and this factor may contribute more or exclusively to the facilitating effect of food restriction on spatial performance in aged rats than the restriction as such (Fitting et al., 2008).

All of these factors may also contribute to a higher variability in individual responses of experimental animals in a task and age dependent manner because of different stress sensitivity and differences in the induction of motoric activity (Blokland and Raaijmakers, 1993; Gallagher et al., 2006; Bizon et al., 2009).

A long-lasting *ad libitum* feeding with a low caloric diet may circumvent these disadvantages. Pitsikas et al. (1990, 1991) found some facilitating effects in the water maze and passive avoidance performance in aged rats after a life long feeding regimen with a low caloric diet, however, possible effects in food rewarded tasks are not studied. Therefore we tested the effect of *ad libitum* feeding of a standard and a energy reduced diet, both providing the same components but in different amounts, on motivation and cognitive abilities in a food rewarded spatial task. The focus was especially on age dependent handling of the different diets and on the homogeneity of individual behavioral performance.

## Materials and Methods

Aged (17–18 months) adult (6–7 months), and young (3–4 months) male Sprague–Dawley rats, bred and maintained in the Core Unit of Biomedical Research, Division of Laboratory Animal Science and Genetics, Medical University of Vienna were used. Rats were housed in groups of three in standard Makrolon cages filled with autoclaved wood chips (temperature: 22 ± 2°C; humidity: 55 ± 5%; 12 h artificial light/12 h dark cycle: light on at 7:00 a.m.). The study was carried out according to the guidelines of the Ethics committee, Medical University of Vienna, and were approved by the Federal Ministry of Education, Science and Culture, Austria.

### Holeboard

The holeboard (1 m × 1 m) manufactured of black plastic surrounded by translucent plexiglass walls. The walls were equipped with proximal spatial cues, surrounding room structures served as distal cues. Four out of 16 regularly arranged holes (diameter and depth 7 cm) were baited (dustless precision pellets, 45 mg, Bioserv^®^, Flemington, NJ, United States) with the pattern of baited holes remained the same during the entire test. A second board below the first was provided with scattered food pellets to avoid olfactory orientation. Ten minutes handling sessions per day for 4 days prior to the experiment made the rats familiar to the experimenter. The following 2 days animals were habituated to the hole-board free exploration of the maze for 15 min each day with access to food pellets. Controlled food restriction over these 6 days reduced the weight of the rats to reach 85% of its initial body weight, which was maintained over training. Tap water was given *ad libitum*. Training consisted of 3 days (five trials on day 1, four trials on day 2, and a retention trial at day 3) with an intertrial interval of 20 min for individual rats. Trial duration was 120 s or until all four pellets were eaten. The apparatus was cleaned with 0.1% Incidin between trials in order to remove odor cues of individual rats. Performance of the rats was recorded by a video camera and stored on a computer. The hole visits and removals of pellets were noted for each trial. In order to compare rats with similar levels of motivation, rats with less than 40 hole visits in total over the 10 trials were excluded from the analysis. This number was chosen because animals with 40 hole visits have the chance to find all four pellets during each of the 10 trials. This ensures that behavioral performance is related to cognition and not motoric activity (Smidak et al., 2017; Kristofova et al., 2018).

Reference memory errors were noted as the number of visits to the unbaited holes. Reference Memory Index (RMI) was calculated using the formula (first+revisits of baited holes)/total visits of all holes. All behavioral training/testing was performed during the light phase of the light–dark cycle.

#### Age Groups

We chosed young and adult groups because first are not fully socially mature and develop no social dominance structure (Adams and Boice, 1989) whereas adult rats develop social dominance and have reached full musculoskeletal maturity (Adams and Boice, 1989; Sengupta, 2011). Aged Sprague–Dawley rats are sensitive to tumor development with a mean life expectancy of 22–26 months (Nakazawa et al., 2001) which represents a senile state. Therefore we chosed slightly younger rats as aged rats. Further, we separated these groups according to the different time periods of feeding the different diets. A timetable of the feeding regimen is given in **Table 1**.

**Table 1:** Timetable of the different feeding regimens.

Age (months)	Young	Adult	Aged
2	Standard	Standard	Standard
3–4	Low energy or standard (test)	Low energy	Standard
6–7		Low energy (test)	Standard (test)
13–18			Low energy (test)

*Test: holeboard test*.

### Diets

Standard (R/M-H), and low energy (R/M-H Ered II) diets from (ssniff^®^, Soest, Germany) and tap water was available *ad libitum* during housing. Food contents are given in **Table 2** according to the informations provided by the manufacturer. Rats were fed with the standard diet up to 10–12 weeks and than the low energy diet was given. In case of test and retest of the aged rats standard diet was applied until reaching adulthood, rats were then tested and subsequently low energy diet was given until the retest as aged rats.

**Table 2:** Composition of the standard diet (STD) and the low energy diet (LED) according to the informations provided by the manufacturer.

Energy (MJ/kg)	STD	LED	Fatty acids (%)	STD	LED	Amino acids (%)	STD	LED	Vitamins (per kg)	STD	LED
Gross energy	16.3	16.6	C14:0	0.01	0.01	Lysine	1.10	0.80	Vitamin A	25.000 IU	15.000 IU
Metabolizable	12.8	8.9	C16:0	0.47	0.54	Methionine	0.35	0.35	Vitamin D3	1000 IU	1000 IU
From Carbohydrates (%)	58.0	46.0	C16:1	0.01	0.02	Met+Cys	0.70	0.63	Vitamin E	135 mg	125 mg
From fat (%)	9.0	12.0	C18:0	0.08	0.10	Threonine	0.68	0.64	Vitamin K	20 mg	5 mg
From protein (%)	33.0	42.0	C18.1	0.62	0.54	Trypthophan	0.25	0.20	Thiamin (B1)	86 mg	17 mg
**Cruide Nutrients (%)**			C18.2	1.79	1.57	Arginine	1.16	0.89	Riboflavin (B2)	32 mg	25 mg
Dry matter	87.8	89.3	C18:3	0.23	0.34	Histidine	0.45	0.36	Pyridoxine (B6)	32 mg	21 mg
Protein (Nx6.25)	19.0	16.5	C20:0	0.01	0.01	Valine	0.90	0.83	Cobolamin (B12)	150 μg	100 μg
Fat	3.3	3.2	C20:1	0.02	0.01	Isoleucine	0.78	0.69	Nicotinic acid	165 mg	123 mg
Fiber	4.9	15.5	C20:5	–	–	Leucine	1.33	1.21	Pantothenic acid	62 mg	45 mg
Ash	6.4	7.2	C22:6	–	–	Phenylalanine	0.87	0.74	Folic acid	10 mg	8 mg
N free extracts	54.2	46.9	**Trace elements (mg/kg)**			Phe+Tyr	1.47	1.18	Biotin	730 μg	715 μg
Starch	36.6	19.3	Iron	176	294	Glycine	0.82	0.75	Choline-Cholide	3.000 mg	2.900 mg
Sugar	4.7	4.5	Manganese	69	66	Glutamic acid	3.97	2.79	Inositol	100 mg	100 mg
**Minerals (%)**			Zin	94	89	Aspartic acid	1.66	1.43			
Calcium	1.00	1.10	Copper	16	17	Proline	1.28	1.07			
Phosphorus	0.70	0.60	Iodine	2.2	2.1	Alanine	0.81	0.81			
Sodium	0.24	0.20	Selenium	0.3	0.4	Serine	0.92	0.73			
Magnesium	0.23	0.25	Cobalt	0.1	2.1						
Potassium	0.92	1.72									

### Statistics

Adult rats are from two large cohorts, one fed with standard diet (*n* = 162) and one with low energy diet (*n* = 120), the standard diet cohort was provided with low energy diet from months 13 to 18 of age and then tested (**Table 1**). After 18 months 127 rat remained from this cohort. For the multigroup comparison of the performance of all rats under standard diet with the same rats aged under low energy diet, 10 rats from the larger cohorts of were randomly selected (Microsoft Excel 2007 random selection procedure) in order to yield similar sample sizes as for the young rats. Differences in behavior, body weight and food intake were tested with the general linear model for repeated measures with trial/time and diet as factors. Differences in retention performance in the holeboard were tested with one way ANOVA. Border of significance was set at *p* ≤ 0.05.

## Results

### Behavioral Performance

#### Reference Memory and Hole Visits

By comparing all groups (*n* = 10, each) we found an overall difference in the reference memory indices [*F*_(4,45)_ = 47.28, *p* < 0.001], with higher indices of adult low energy diet compared to adult standard diet (*p* < 0.001) and aged low energy diet (*p* = 0.005), whereas there was no difference as compared to young low energy and standard diet rats (*p* = 1, each). Adult standard diet animals performed worse as compared to all other groups (*p* < 0.001, each). The aged low energy diet group performed less well when compared to the young low energy diet group (*p* = 0.049) and adult low energy rats (*p* = 0.005) but not compared to the young standard diet group (*p* = 0.211).

An overall difference between groups could be determined also in the total numbers of hole visits [*F*_(4,45)_ = 16.62, *p* < 0.001], with higher numbers of adult low energy diet as compared to adult standard diet rats (*p* < 0.001), but not when compared to the other groups (*p* = 0.2–1). Adult standard diet rats show less visits as compared to all other groups (*p* < 0.001, each). Aged and young low energy diet animals and young standard diet rats show no difference compared to all groups except the adult standard diet group (*p* = 0.3–1).

**FIGURE 1:**
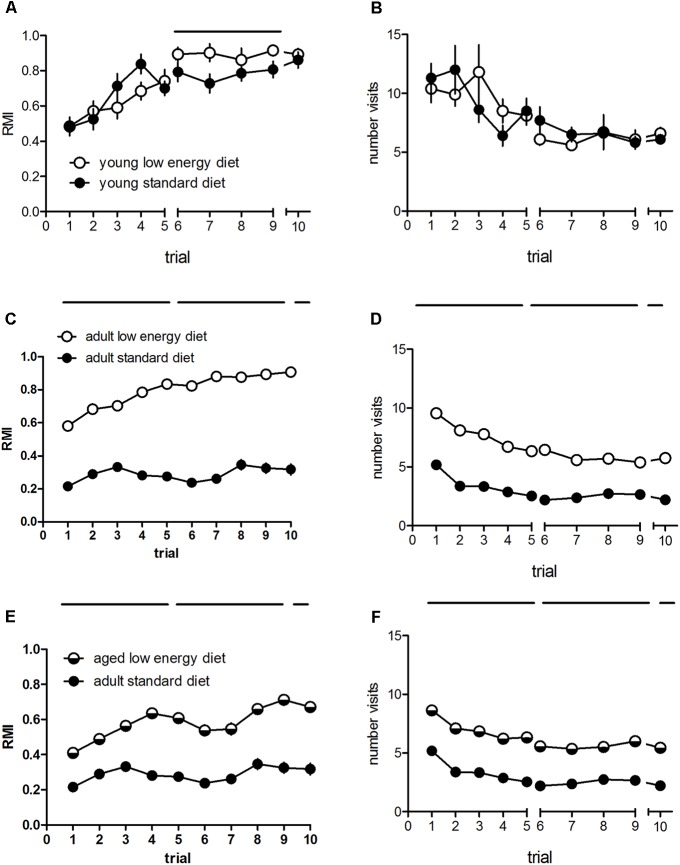
Reference memory indices **(A)** and numbers of total hole visits **(B)** of young (*n* = 10, each) and adult rats under standard (*n* = 162) and low energy diet (*n* = 120). **(C,D)** Reference memory indices **(E)** and total hole visits **(F)** of adult rats under standard diet (162) and the same rats when aged under low energy diet (*n* = 127). Horizontal bars indicate statistically significant differences between groups. Given are the means and standard errors of means.

Some groups were compared in more detail. There were no significant effects of diet on the RMI in young rats [*F*_(1,18)_ = 0.87, *p* = 0.36; **Figure 1A**], and also no diet × trial interaction [*F*_(9,162)_ = 1.19, *p* = 0.31] whereas training significantly affects RMI [*F*_(9,162)_ = 15.6, *p* < 0.001], indicating that learning has taken place. The comparison of the entire cohorts of the adult rats revealed a significant diet effect [*F*_(1,280)_ = 345.05, *p* < 0.001], a significant trial effect [*F*_(9,2520)_ = 30.62, *p* < 0.001] and a significant diet × trial interaction [*F*_(9,2520)_ = 16.12, *p* < 0.001] when reference memory indices are compared. Adult rats fed with the standard diet show significantly lower reference memory indices as compared to adult rats fed with the low energy diet (**Figure 1C**). In contrast, when we compare adult performance with the performance of the same rats when aged we found a significant diet effect upon RMI [*F*_(1,287)_ = 88.5, *p* < 0.001; **Figure 1E**], with better performance of the aged rats. Further, there was a significant trial effect [*F*_(9,2583)_ = 23.95, *p* < 0.001] and a diet trial interaction [*F*_(9,2583)_ = 6.72, *p* < 0.001]. Young rats show no diet effect on hole visits [*F*_(1,18)_ = 0.00, *p* = 0.96; **Figure 1B**] no diet × visit interaction [*F*_(9,162)_ = 1.19, *p* = 0.31] but a significant trial effect [*F*_(9,162)_ = 7.15, *p* < 0.001], whereas adult rats differed significantly (**Figure 1D**), with higher numbers of adult rats fed with the low energy diet as compared to adults fed with the standard diet [*F*_(1,280)_ = 222.40, *p* < 0.001]. There was a significant trial effect [*F*_(9,2520)_ = 67.28, *p* < 0.001] and a significant diet × trial interaction [*F*_(9,2520)_ = 3.67, *p* < 0.001]. There was a significant diet effect on hole visits between adult rats under standard diet and the same rats when aged under low diet [*F*_(1,287)_ = 137.80, *p* < 0.001; **Figure 1F**] as well as a trial effect [*F*_(9,2583)_ = 45.77, *p* < 0.001] and no diet × trial interaction [*F*_(9,2583)_ = 1.19, *p* = 0.29].

When different training phases are compared with aquisition phase 1 (day 1) and 2 (day 2) and retention (day 3) we found a significant difference between diet groups in young rats during day 2 [*F*_(1,18)_ = 8.9, *p* = 0.008] but not during day 1 [*F*_(1,18)_ = 0.59, *p* = 0.45] and retention (*t* = 0.57, df = 18, *p* = 0.58), whereas hole visits were not different during any phase [day 1: *F*_(1,18)_ = 0.12, *p* = 0.74; day 2: *F*_(1,18)_ = 0.85, *p* = 0.37; retention: *t* = 0.80, df = 18, *p* = 0.43]. There was a diet effect between adult standard and low energy diet groups regarding the reference memory indices during acquisition phase 1 [*F*_(1,280)_ = 299.36, *p* < 0.001] and phase 2 [*F*_(1,280)_ = 331.40, *p* < 0.001] and during the retention phase (*t* = -14.66, df = 280, *p* < 0.001). Hole visits for these groups differed during any phase: acquisition phase 1 [*F*_(1,280)_ = 212.32, *p* < 0.001] and phase 2 [*F*_(1,280)_ = 178.17, *p* < 0.001] and during the retention phase (*t* = -11.42, df = 280, *p* < 0.001). Between adult standard diet and aged lower energy diet we found signficant differences in RMI at all phases [day 1: *F*_(1,287)_ = 84.15, *p* < 0.001; day 2: *F*_(1,287)_ = 76.23, *p* < 0.001; retention: *t* = -7.89, df = 287, *p* < 0.001]. Also the number of hole visits between these groups differed during all phases [day 1: *F*_(1,287)_ = 116.97, *p* < 0.001; day 2: *F*_(1,287)_ = 127.19, *p* < 0.001; retention: *t* = -9.7, df = 287, *p* < 0.001].

The difference in diet related cognitive performance, however, cannot merely explained by the difference in hole visits, since a significant difference between RMI can still be found when the sum of hole visits is included as controlling covariate [*F*_(1,286)_ = 11.84, *p* = 0.001]. The sum of hole visits differ [*F*_(1,286)_ = 90.23, *p* < 0.001] the effect size (partial eta squared) is higher for hole visits (0.24) than for RMI (0.04).

#### Behavioral Variability

**FIGURE 2:**
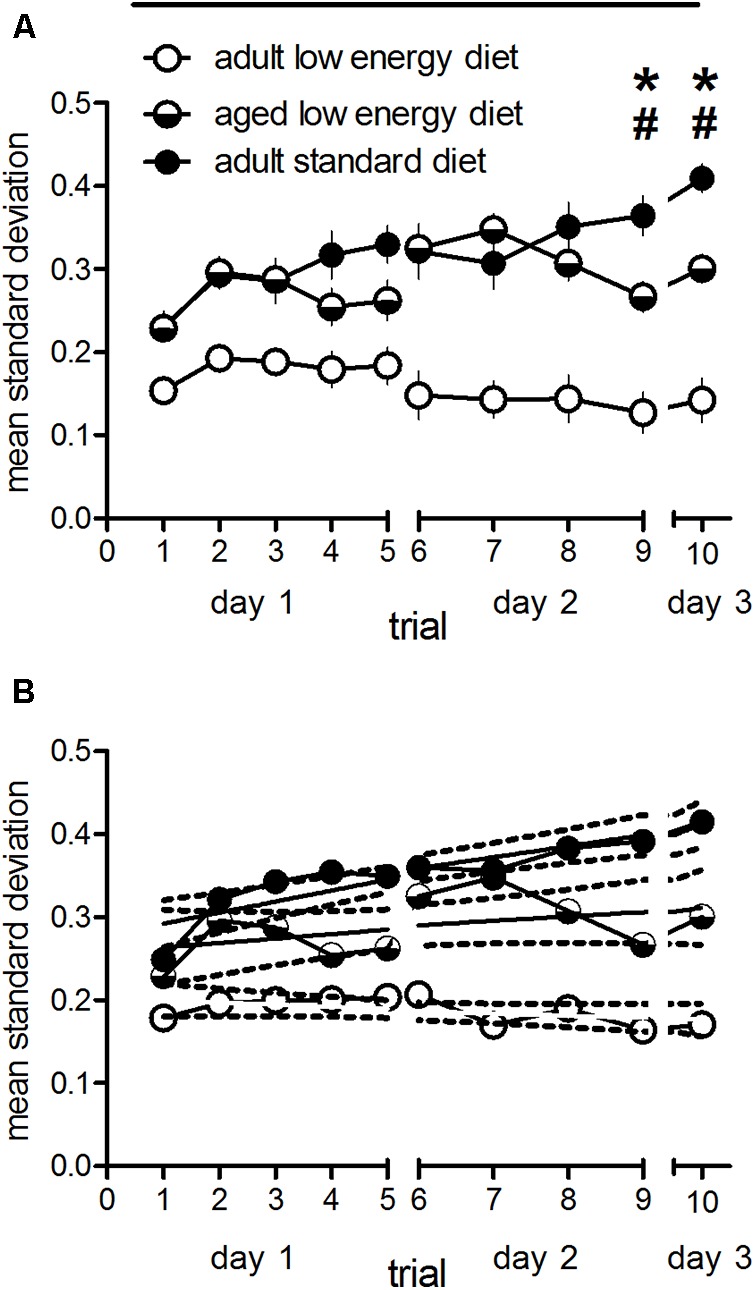
Standard deviations of the reference memory indices of subdivisions (*n* = 10, each, *n* = 7 for one sample of aged low energy diet rats; **(A)** and linear regression with 95% confidence intervals (broken lines) of the mean standard deviations **(B)** of adult animals fed with standard and adult and aged rats under low energy diet. Horizontal bars indicate statistically significant differences between groups over the entire training. Asterisks and hash marks indicate trial specific differences between adult standard and low energy diet fed rats and adult standard and aged low energy diet fed rats, respectively. Given are the means and standard errors of means.

In order to analyse effects of the different feeding on behavioral variability we divided the cohorts into groups of 10 animals (to make it comparative to the other samples) and calculated the standard deviation of each of these groups/trial. Thus, we have 16 samples for the adult standard diet group (*n* = 162), 12 for the adult low energy diet food (*n* = 120) and 13 (sample 13 only containing seven animals) for the aged low energy diet food (*n* = 127), the total sample diminished as compared to the adult cohort by natural death and disease). In **Figure 2A** the mean values of standard deviations for these groups are given. There is a significant overall effect between groups [*F*_(2,38)_ = 25.20, *p* < 0.001] with a significant trial effect [*F*_(9,342)_ = 4.24, *p* < 0.001] and a diet × trial interaction [*F*_(18,342)_ = 4.87, *p* < 0.001]. *Post hoc* test over all trials revealed significantly lower standard deviations in low energy diet fed adult animals as compared to adult and aged standard diet fed animals (*p* < 0.001, each), whereas the two latter groups did not differ (*p* = 0.324). However, when trial specific *post hoc* tests (Bonferroni) were performed, standard diet and low energy diet adult animals showed significant differences in standard deviations during trial 2–10 (*p* < 0.05 at trial 2 and 3, *p* < 0.001 at trial 4–10). Low energy diet fed adult animals differed during trial 2 and 3 (*p* < 0.05) and trial 6–10 (*p* < 0.01) as compared to low energy diet fed aged rats. Interestingly, standard diet fed adult rats showed significantly higher standard deviations at trial 9 (*p* < 0.05) and 10 (*p* < 0.01) as compared as aged rats under low energy diet. Trial 9 reflects learning, whereas trial 10 reflects memory performance. Thus, the mean standard deviation increases over trials in standard food fed adult rats, but not in low energy food fed adult and aged rats indicated by significant differences in slopes over trials between these groups [*F*_(2,24)_ = 8.96, *p* < 0.01; **Figure 2B**]. The slope is significantly different from zero in the standard diet fed adult animals [*F*_(1,8)_ = 33.36, *p* = 0.0004] but not in the low energy diet fed adult [*F*_(1,8)_ = 2.75, *p* = 0.14] and aged rats [*F*_(1,8)_ = 2.12, *p* = 0.18].

#### Body Weights and Food Consumption

**FIGURE 3:**
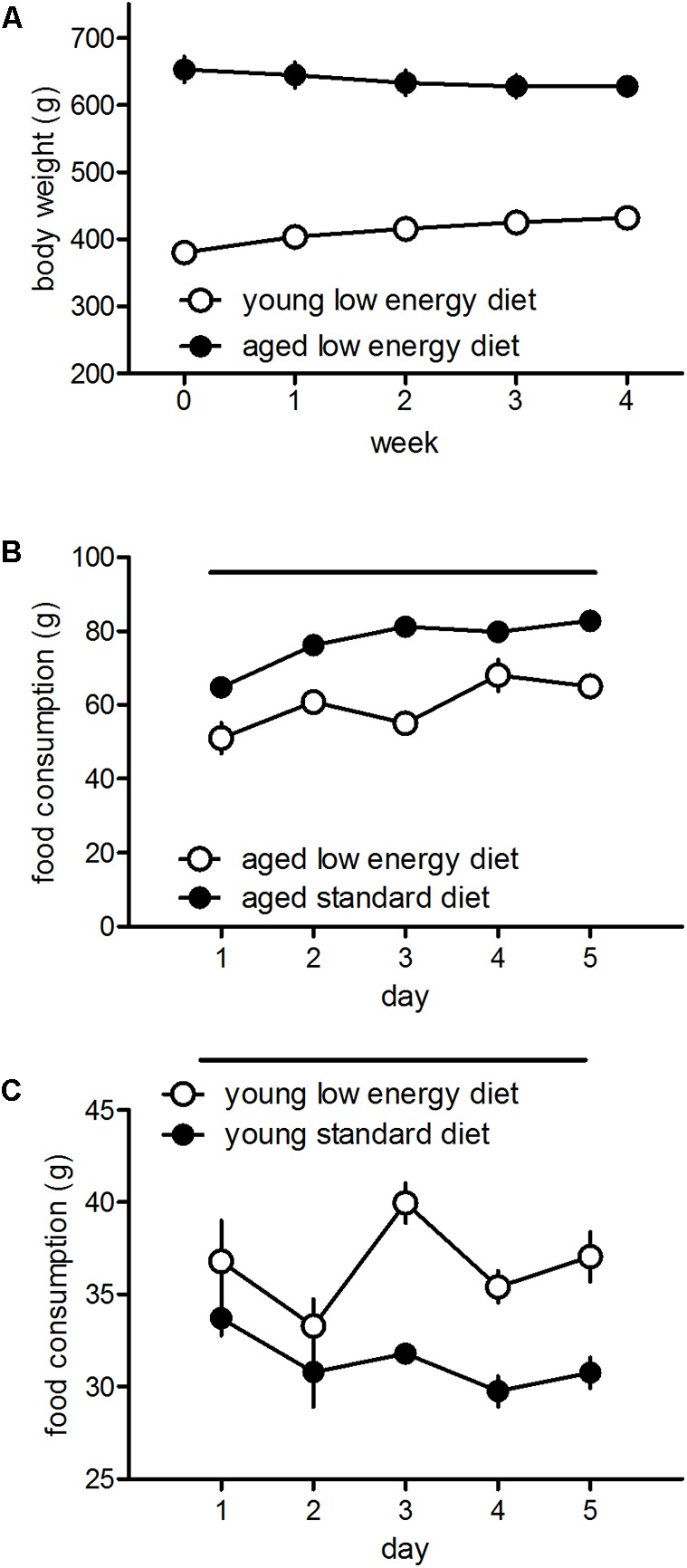
Development of body weights over 4 weeks of the aged (*n* = 14) and young (*n* = 20) rats fed with the low energy diet **(A)** and food intake over 5 days of the aged (**B**, five cages with three rats each) and young (**C**, four cages with three rats each) rats fed with the low energy or standard diet. Horizontal bars indicate statistically significant differences between groups. Given are the means and standard errors of means.

Young and aged rats fed with the low energy diet differ in their food consumption and body weight. Aged rats show a reduction of body weights [*F*_(4,13)_ = 8.17, *p* < 0.001] whereas young rats show an increase [*F*_(4,19)_ = 100.4, *p* < 0.001] over 4 weeks (**Figure 3A**). Aged rats show a reduced food intake of low energy diet food as compared to standard diet intake [*F*_(1,8)_ = 108.39, *p* < 0.001; **Figure 3B**], whereas young rats show elevated food intake when fed with the low energy diet [*F*_(1,6)_ = 68.03, *p* = 0.0002; **Figure 3C**].

## Discussion

We found large differences in spatial learning and memory between rats fed with moderately different diets in an age dependent manner. Whereas learning but not memory was only slightly improved in young rats fed with a low energy diet, both learning and memory was improved in adult and aged rats. The largest effect, however, could be noted for motivation indicated by the number of hole visits (Post et al., 2011) in the aged rats, which was unaffected in young rats. The effect of caloric restriction on spatial learning seems to be task dependent, and depends on the kind of delivering fewer calories. Caloric restriction does not prevent cognitive aging in rats when tested in a water maze (Markowska, 1999), whereas feeding of a standard or hypocaloric diet provided *ad libitum* improved some cognitive deficiencies in aged rats in the latter group (Pitsikas et al., 1990), although learning rather than memory was affected. Stewart et al. (1989) found minor effects of food restriction in the radial maze mostly at younger ages and a small improvement of restricition of aged rats in the water maze. In a direct comparison of food rewarded maze and water maze performance it was found that food restricted young rats performed better than freely fed rats in both the food rewarded and the water maze task (Kant et al., 1988). A corresponding modulating factor may be stress, because it is known that forced food restriction increases the level of the stress hormone corticosterone in rats (Kant et al., 1988; Heiderstadt et al., 2000; Uzakov et al., 2005; MacDonald et al., 2014) which at the long-term may cause negative effects on hippocampal neurons, and therefore counteract the beneficial effects of the low energy diet on hippocampal dependent spatial cognition. This may be relevant especially when animals are tested in the water maze which additionally increases the stress levels as compared to dry mazes. Food restriction increased corticosterone levels before swimming and impaired water maze performance (Chu et al., 2003). However, Andrade et al. (2002) found increased synapse densities in the molecular layer of dentate gyrus and reduced segments of dendrictic arborizations of granule cells in food restricted rats as compared to controls and no differences in the water maze performance, whereas passive avoidance performance was impaired in food restricted rats. Water maze experiments were performed at relatively cold water (21°C), which heavily affects water maze performance (Salehi et al., 2010). Staples et al. (2017), however, found reduced dentate gyrus neurogenesis and granule cell density in forced food restricted rats, when restriction started at an age of 2 months, whereas Lee et al. (2000) found increased neurogenesis after dietary restriction started at an age of 3 months and lasting for 3 months in rats.

Self selection of food and food intake seems to be a crucial factor to adequately regulate body composition. Rats initially fed with a high-fat diet reduced the intake of this diet when allowed to choose between this and a lower energy diet developing more fat free body mass (Azzout-Marniche et al., 2016). Although aged rats in the present study could not choose, they reduced their food intake of the lower energy diet in contrast to the young rats which probably need to compensate for the lower availability of food contents in the energy reduced diet. Age related differences in diet intake are known; Veyrat-Durebex et al. (1998) found decreased fat and protein rich diets intake in male rats from an age of 4 months to 20 months while the intake of a standard diet increased and remained stable during aging. The present study show large effects of moderately different diets on motivation and cognitive performance especially in aged rats. Although, motivation to explore the maze and to chase for food pellets is mostly affected by the difference in diets as indicated by the statistical effect size, there is also a statistical significant effect upon cognition. However, cognition is not affected in all rats to the same extent as indicated by the large variability of spatial reference memory indices in adult rats under the standard diet. This high variance can be reduced by a lower energy diet with only moderate differences in food composition compared to the standard diet even in the same animals when aged and only in training phases crucial for learning and memory, which may be of interest for increasing the statistical reliability in aging studies.

## Author Contributions

GL, HH, and VK developed the concept. JM, DF, and AH performed the experiments. VK analyzed the data. GL and VK wrote the manuscript. All authors read and approved the manuscript.

## Conflict of Interest Statement

The authors declare that the research was conducted in the absence of any commercial or financial relationships that could be construed as a potential conflict of interest.
